# Classification systems for assessing acute muscle injuries: a retrospective comparison of inter-reader agreements

**DOI:** 10.1007/s00256-025-04988-1

**Published:** 2025-08-13

**Authors:** Oliver A. Binkert, Christian W. A. Pfirrmann, Sonja Fierstra, Kai Higashigaito, Andrea B. Rosskopf

**Affiliations:** 1 Faculty of Medicine, University of Zurich, Zurich, Switzerland; 2 Medical Radiological Institute (MRI) Zurich, Schulthess Clinic, Lengghalde 2, CH-8008 Zurich, Switzerland

**Keywords:** MR imaging, Diagnostic, Muscle injury, Edema, Classification

## Abstract

**Objectives:**

The purpose of this study is to compare three commonly used classification systems for MRI grading of acute muscle injury concerning their inter-reader reliability.

**Methods:**

Ethical committee approval was obtained. Inclusion criteria comprised patients with acute muscle injury, age ≥ 18 years, and signed informed consent. MR examinations were evaluated by four independent musculoskeletal radiologists. Muscle injuries were graded according to the British Athletics Muscle Injury Classification (BAMIC), the Munich Consensus Injury Classification (MCIC), and the Chan et al. Injury Classification (CIC). Inter-reader reliability was quantified with Fleiss’ Kappa (*κ*) and associated 95% confidence interval (CI).

**Results:**

One hundred eleven acute muscle injuries in 110 patients (84% males) were assessed. Injured muscle groups included 85 thigh injuries (44 hamstrings, 41 non-hamstrings), 19 lower leg injuries, and 7 injuries in other locations. *κ* values (CI) were 0.506 (0.499, 0.514) for BAMIC, 0.566 (0.549, 0.584) for MCIC, and 0.306 (0.302, 0.311) for CIC. The highest reproducibility was seen for non-hamstring injuries in the thigh using MCIC 0.749 (0.720, 0.777), the lowest for lower leg injuries using CIC 0.199 (0.185, 0.213). Injury severity showed greater reproducibility (*κ* = 0.594–0.696) than the location of the injury within the muscle (*κ* = 0.349–0.576).

**Conclusions:**

The MCIC and BAMIC demonstrate moderate inter-reader reliability, whereas the CIC demonstrates fair inter-reader reliability. The challenge with the classifications is the reproducibility of localizing the injury anatomically within the muscle, rather than classifying injury severity. Non-hamstring thigh injuries were most reproducible with MCIC, while lower leg injuries were least reproducible with CIC.

**Supplementary Information:**

The online version contains supplementary material available at 10.1007/s00256-025-04988-1.

## Introduction

Acute muscle injuries are of significant importance in musculoskeletal (MSK) radiology due to their high prevalence, particularly during sporting activities. Such injuries often result from non-contact, explosive movements such as sprinting, stretching, or jumping [1, 2]. Sports frequently associated with such injuries include football, Australian football (AFL), American football (NFL), rugby, and track and field disciplines [2, 3].

The most prevalent type of injury is a muscle strain resulting from indirect trauma. These injuries primarily affect the lower extremities, with the hamstring muscle complex being the most involved [4, 5]. In the thigh, second most common muscle strains occur in the rectus femoris muscle, in soccer players most often at the myotendinous junction and primarily caused by kicking rather than sprinting [6, 7]. In some cases, a specific type of rectus femoris injury known as a degloving injury is observed, in which the inner bipennate portion of the rectus femoris separates from the outer unipennate portion [8]. Risk factors for indirect muscle injuries include eccentric contraction, a high proportion of fast-twitch type II fibers, sudden changes in muscle function, muscle imbalances, and muscles that span multiple joints (e.g., biceps femoris, rectus femoris, gastrocnemius) [9]. Muscle injuries can lead to significant time loss from competition and a heightened risk of re-injury. In many cases, clinical examination alone is insufficient to accurately assess the injury or predict return-to-play timing [9, 10]. Imaging techniques are therefore employed to provide additional diagnostic and prognostic information. Both ultrasound and MRI play important roles in the assessment of muscle injuries. However, MRI is considered the imaging gold standard for the evaluation of acute muscle injuries due to its superior soft tissue contrast and ability to delineate injury extent [11, 12]. Numerous studies have demonstrated that return-to-play timelines following muscle injuries are influenced by a multifactorial set of variables—including the athlete’s physical condition, as well as clinical and functional assessments—and that the isolated prognostic value of MRI findings remains limited [13–15]. Nevertheless, MRI retains a crucial role in delineating the extent of the injury, information that is essential for both the medical team and the athlete. To facilitate a standardized understanding of injury severity and localization, sports medicine physicians frequently ask for specific MRI-based classification systems. MRI-based assessment relies on classification systems in which radiologists grade the injuries based on various anatomical parameters [9, 16]. The classification grade can influence key clinical decisions, such as the degree of rest and the estimated return-to-play time, which is particularly relevant in athletes like soccer players [17]. Hence, the development of a reliable and reproducible classification system is essential. Given the complexity of muscle injuries, multiple parameters may be included in a grading system, leading to the existence of several classification methods for the same lesion. A major limitation of these systems is inconsistent reliability, as grading can vary between radiologists. Therefore, inter-reader reliability is a crucial feature in determining the clinical utility of a classification system.

Sports medicine physicians within our region most often request one of these commonly used MRI-based classification systems:

- The British Athletics Muscle Injury Classification (BAMIC, 2014) [18]
- The Munich Injury Consensus Classification (2013) [19]
- The Chan et al. Injury Classification (2012) [20]

This study aims to evaluate and compare the inter-reader reliability of these three MRI-based muscle injury classification systems to determine which demonstrates the highest diagnostic consistency and clinical utility. This is the first study to assess inter-reader reliability for muscle injuries in the upper and lower extremities, including both hamstring and non-hamstring injuries.

## Material and methods

The local ethics committee approved the study protocol (BASEC 2024-00777) and defined the patient selection period from December 5, 2020, to June 11, 2024. Inclusion criteria were age ≥ 18 years old, written informed consent for scientific research, MRI of the upper or lower extremity, diagnosis of acute muscle injury in the MRI report, and a complete MRI examination comprising at least two fluid-sensitive sequences (with at least one transverse plane and one longitudinal plane) and one T1-weighted turbo spin-echo (TSE) sequence.

**Fig. 1:**
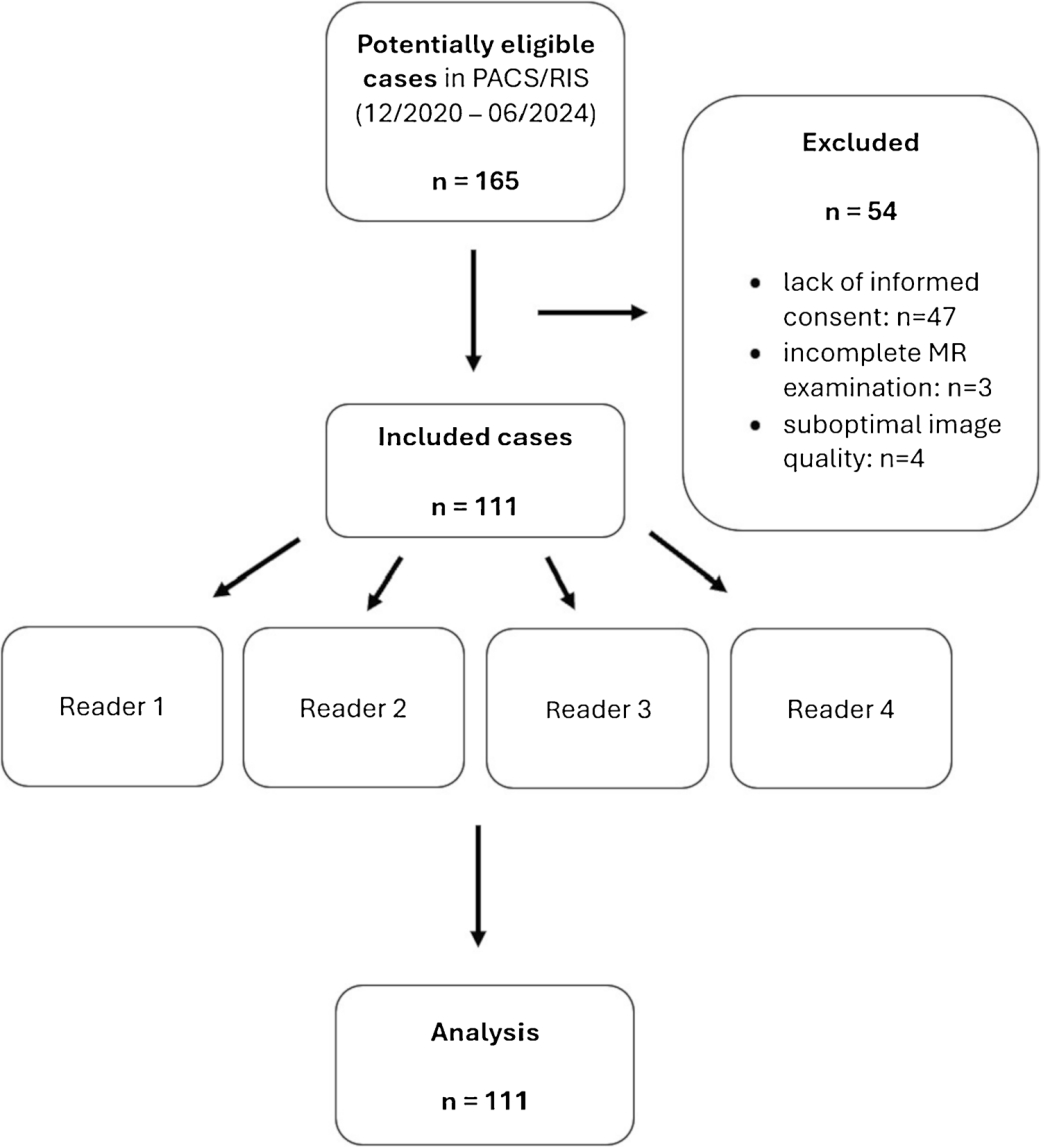
Study flowchart

Exclusion criteria were lack of research consent, incomplete MRI examinations, or suboptimal image quality (e.g., due to motion artifacts). A detailed study flowchart is presented in Fig. 1.

### MRI images

All patients were scanned in supine position in one of these institutional scanners: 1.5 Tesla (Sola/Avanto/Aera; Siemens Healthcare, Erlangen, Germany) or 3.0 Tesla (Vida/Skyra; Siemens Healthcare, Erlangen, Germany) depending on scanner availability at the time of referral (the institutional routine protocol for the 3 Tesla-scanner (VIDA) can be found in the supplemental material: Table S1). MR protocol included at least two fluid-sensitive sequences (with at least one transverse plane and at least one longitudinal plane) and one T1-weighted TSE sequence.

### Evaluation of MR examination

All images were independently reviewed by four fellowship-trained musculoskeletal radiologists: S.F. (13 years of experience), K.H. (12 years), A.B.R. (19 years), and C.W.A.P (27 years). Readers were blinded to the original MRI reports and to each other’s assessments. The injured muscle was identified prior to the scoring of the classification systems. In cases with more than one lesion, the most severe lesion was scored. Image evaluation was conducted using REDCap (version 13.5.4; Vanderbilt University, Nashville, TN, USA), according to each of the following three MR classification systems.

### Classification systems

**Fig. 2:**
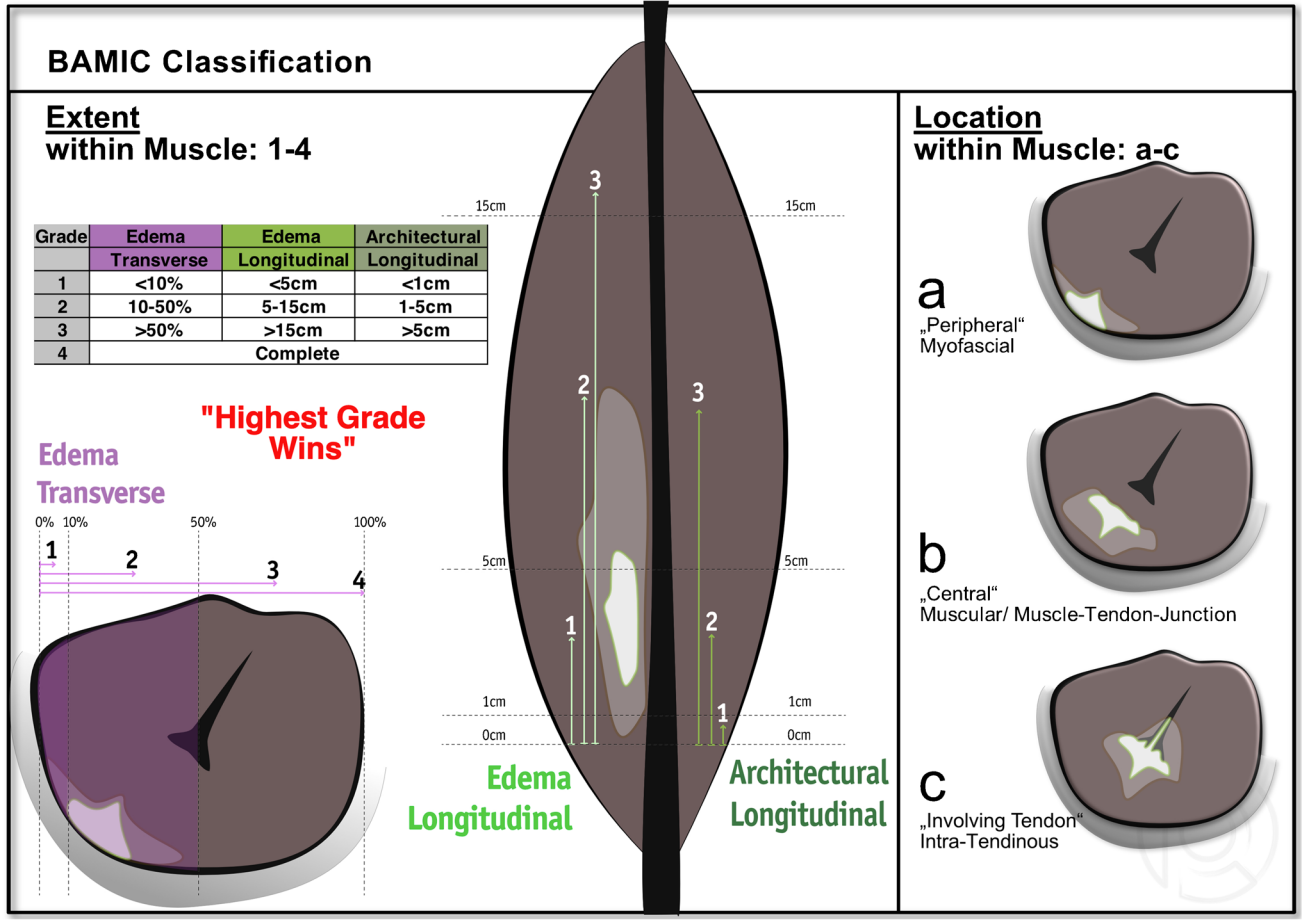
Scheme of the British Athletics Muscle Injury Classification (BAMIC)
- The Munich Consensus Injury Classification (MCIC; 2013) [19] (focusing on injury types 3 and 4), see details in Fig. 3. Types 1 and 2 were excluded, as they rely primarily on clinical parameters and cannot be diagnosed using MRI alone.

- The British Athletics Muscle Injury Classification (BAMIC, 2014) [18], see details in Fig. 2.

**Fig. 3:**
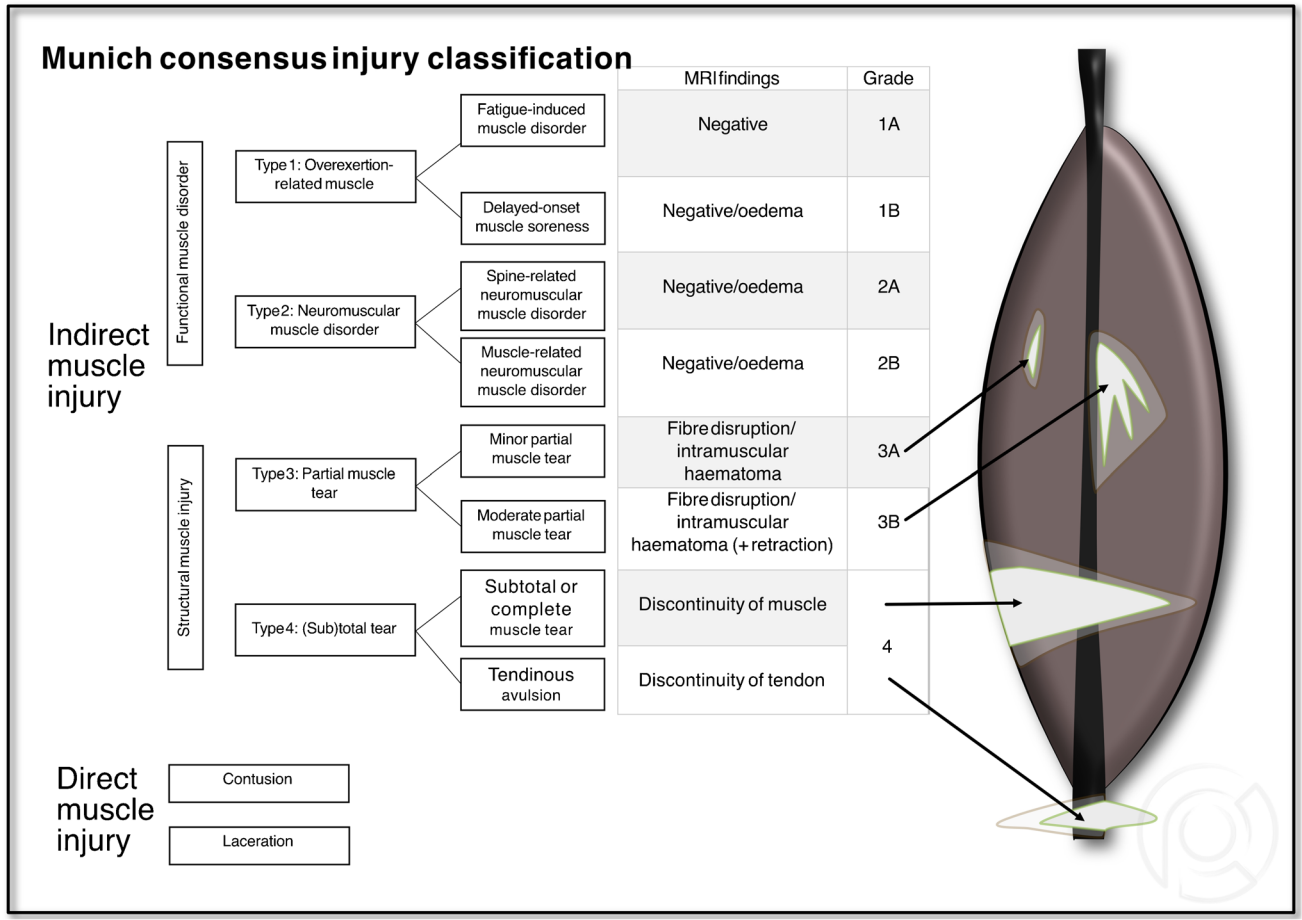
Scheme of the Munich Consensus Injury Classification (MCIC). Note: Only muscle injuries of types 3 and 4 were evaluated in this study
- The Chan et al. Injury Classification (CIC; 2012) [20], see details in Fig. 4.

**Fig. 4:**
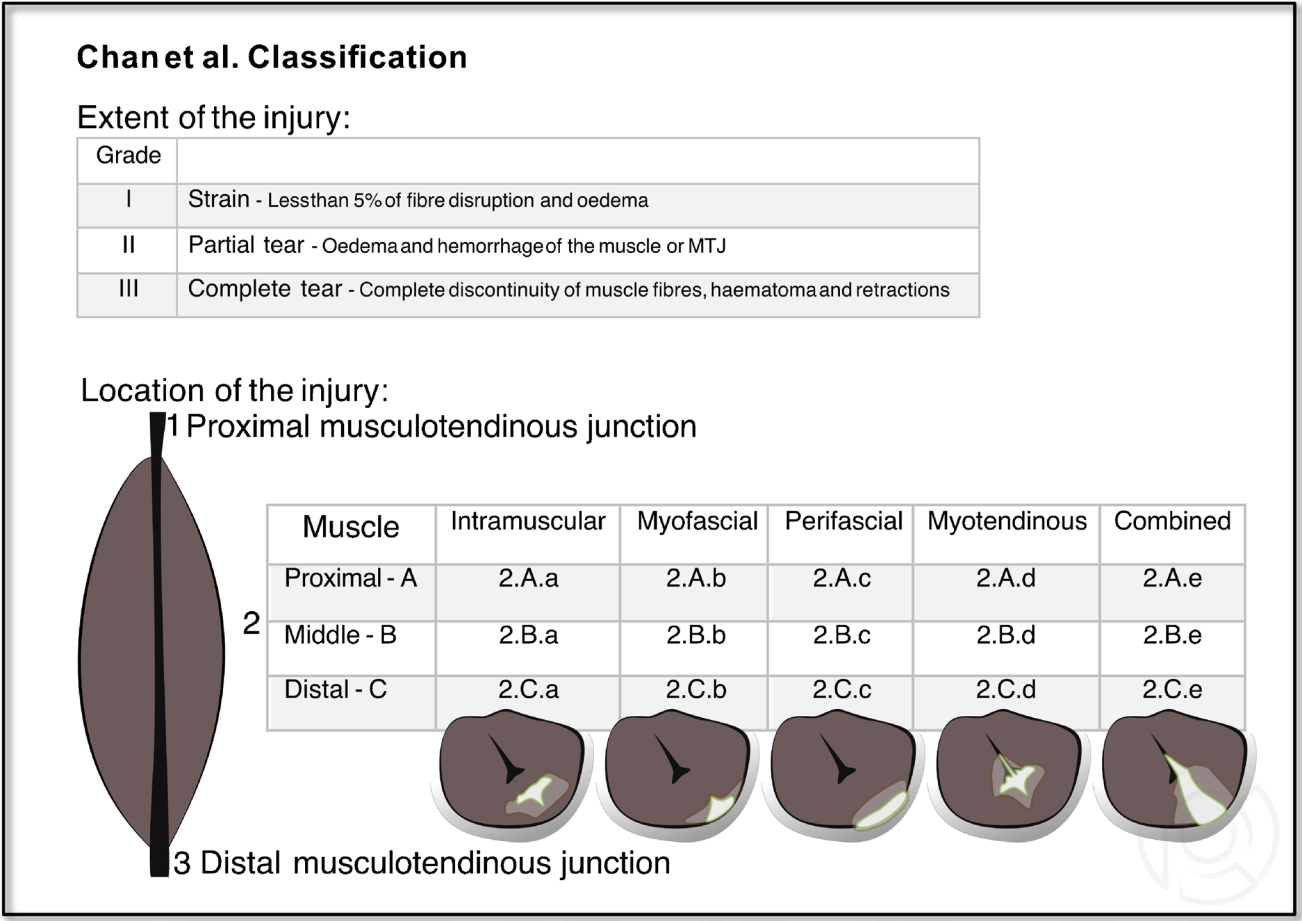
Scheme of the Chan et al. Injury Classification (CIC)

Upon completion of the evaluation, all four readers were asked to rate their confidence in applying each classification system using a 5-point Likert scale: 1 = very uncertain, 2 = somewhat uncertain, 3 = neutral, 4 = somewhat confident, 5 = very confident.

### Statistical analysis

Study data were collected and managed using REDCap® electronic data capture tools (Version 13.5.4; Vanderbilt University, Nashville, TN, USA). The statistical analysis was done by a professional statistician (see “Acknowledgements”).

Inter-reader agreement was quantified with Fleiss’ Kappa (*κ*) and associated 95% confidence interval. No further averaging or other aggregation of k-values was conducted. *κ* values were classified as poor (*κ* < 0), slight (0 ≤ *κ* ≤ 0.20), fair (0.21–0.40), moderate (0.41–0.60), substantial (0.61–0.80), and almost perfect (0.81–1.00) [21]. Patient characteristics were provided as mean and standard deviation, or absolute and relative counts. The analysis was conducted with MATLAB (The MathWorks Inc. (2022b), Natick, MA, USA). *κ* values between rating schemes were compared using *z*-testing. *p*-values below 0.05 were considered statistically significant.

## Results

A total of 111 acute muscle injuries in 110 patients were assessed. The mean age of the patients when the MRI was performed was 31.8 years (± 13.4). Detailed patients’ characteristics can be found in Table 1.

**Table 1:** Patients’/cases’ characteristics (*SD* = standard deviation)

	*n* (%)
All (injuries in 110 patients)	111
Age: mean years (± SD)	31.8 (± 13.4)
Male	93 (83.8)
Right side	60 (54.1)
Hip (M. iliopsoas)	3 (2.7)
Thigh: all	85 (76.6)
Thigh: non-hamstrings	41 (36.9)
M. rectus femoris	21 (18.9)
M. adductor longus	6 (5,4)
M. vastus lateralis	6 (5.4)
M. gracilis	3 (2.7)
M. adductor magnus	2 (1.8)
M. vastus medialis	2 (1.8)
M. vastus intermedius	1 (0.9)
Thigh: hamstrings	44 (39.6)
M. biceps femoris	28 (25.2)
M. semimembranosus	10 (9.0)
M. semitendinosus	6 (5.4)
Lower limb	19 (17.1)
M. gastrocnemius	11 (9.9)
M. soleus	7 (6.3)
M. tibialis anterior	1 (0.9)
Knee (popliteus muscle)	1 (0.9)
Upper limb (M. triceps brachii)	3 (2.7)

### Evaluation of inter-reader agreement

Inter-reader agreement was evaluated across three categories according to the original classification descriptions (see Figs. 2, 3, and 4): (1) injury severity, represented by the initial component of the rating (e.g., BAMIC: **3**a; CIC: **II**.2.A.a); (2) injury location, indicated by the subsequent number(s) or letter(s) (e.g., BAMIC: 3**a**; CIC: II.2. **A.a**); and (3) the overall classification (SUMMARY), combining both severity and location (e.g., BAMIC SUMMARY: **3a**, MCIC SUMMARY: **3A**; CIC SUMMARY: **II.2.A.a**). In contrary to the BAMIC and CIC, MCIC only categorizes injury severity, but not injury location.

*κ* values (95% confidence intervals) for overall agreement were as follows: 0.506 (0.499–0.514) for BAMIC SUMMARY, 0.566 (0.549–0.584) for the MCIC SUMMARY, and 0.306 (0.302–0.311) for the CIC SUMMARY. The highest inter-reader agreement was observed for non-hamstring injuries in the thigh using the MCIC SUMMARY (*κ* = 0.749; 95% CI = 0.720–0.777), while the lowest agreement was found for lower leg injuries using the CIC SUMMARY (*κ* = 0.199; 95% CI = 0.185–0.213). For a detailed overview of the results, refer to Tables 2 and 3.

**Table 2:** Inter-reader agreements: *κ* values (95% confidence intervals)

Variable	Definition	All	Thigh	Lower leg
**BAMIC**_SUMMARY	Combines severity and location, e.g., “2a”	0.506 (0.499, 0.514)	0.504 (0.495, 0.512)	0.482 (0.463, 0.502)
**BAMIC**_Severity	Severity: 1, 2, 3, 4	0.696 (0.684, 0.709)	0.726 (0.711, 0.740)	0.538 (0.506, 0.570)
**BAMIC**_Location	Location: a peripheral, b central, c with tendon	0.524 (0.510, 0.539)	0.491 (0.474, 0.507)	0.631 (0.596, 0.665)
**MCIC**_SUMMARY	Severity: 3 A (minor partial tear), 3B (moderate partial tear), 4 (subtotal or complete muscle tear or tendinous avulsion)	0.566 (0.549, 0.584)	0.619 (0.600, 0.638)	0.489 (0.446, 0.531)
**CIC_**SUMMARY	Combines severity and anatomical and intramuscular location, e.g., “II.2.A.a”	0.306 (0.302, 0.311)	0.319 (0.313, 0.325)	0.199 (0.185, 0.213)
**CIC_**Severity	Severity: I, II, III	0.594 (0.578, 0.609)	0.662 (0.645, 0.680)	0.218 (0.181, 0.255)
**CIC_**Anatomical Location	Anatomical 1 proximal MTJ, 2 muscle, 3 distal MTJ	0.576 (0.561, 0.591)	0.553 (0.535, 0.571)	0.560 (0.518, 0.603)
**CIC_**Muscle Main Location	Only in case of anatomical location “2” (muscle):	0.429 (0.417, 0.442)	0.414 (0.399, 0.429)	0.449 (0.420, 0.478)
	A. Proximal			
	B. Middle			
	C. Distal			
**CIC_**Muscle Sublocation	Only in case of anatomical location “2” (muscle):	0.349 (0339, 0358)	0.364 (0.354, 0.375)	0.280 (0.256, 0.304)
	a. Intramuscular			
	b. Myofascial			
	c. Myofascial/perifascial			
	d. Myotendinous			
	e. Combined			

*BAMIC* British Athletics Muscle Injury Classification, *MCIC* Munich Consensus Injury Classification, *CIC* Chan et al. Injury Classification

**Table 3:** Inter-reader agreements: *κ* values (95% confidence intervals)

Variable	Definition	Thigh – non-hamstrings	Thigh – hamstrings
**BAMIC**_SUMMARY	Combines severity and location, e.g., “2a”	0.501 (0.488, 0.513)	0.489 (0.476, 0.502)
**BAMIC**_Severity	Severity: 1, 2, 3, 4	0.761 (0.739, 0.782)	0.689 (0.670, 0.708)
**BAMIC**_Location	Location: a peripheral, b central, c with tendon	0.474 (0.451, 0.497)	0.471 (0.447, 0.496)
**MCIC**_SUMMARY	Severity: 3 A (minor partial tear), 3B (moderate partial tear), 4 (subtotal or complete muscle tear or tendinous avulsion)	0.749 (0.720, 0.777)	0.587 (0.560, 0.613)
**CIC_**SUMMARY	Combines severity and anatomical and intramuscular location, e.g., “II.2.A.a”	0.354 (0.345, 0.363)	0.263 (0.254, 0.273)
**CIC_**Severity	Severity: I, II, III	0.827 (0.801, 0.854)	0.540 (0.516, 0.564)
**CIC_**Anatomical Location	Anatomical 1 proximal MTJ, 2 muscle, 3 distal MTJ	0.614 (0.589, 0.638)	0.474 (0.444, 0.503)
**CIC_**Muscle Main Location	Only in case of anatomical location “2” (muscle):	0.450 (0.429, 0.470)	0.374 (0.354, 0.395)
	A. Proximal		
	B. Middle		
	C. Distal		
**CIC_**Muscle Sublocation	Only in case of anatomical location “2” (muscle):	0.377 (0.361, 0.392)	0.323 (0.307, 0.340)
	a. Intramuscular		
	b. Myofascial		
	c. Myofascial/perifascial		
	d. Myotendinous		
	e. Combined		

*BAMIC* British Athletics Muscle Injury Classification, *MCIC* Munich Consensus Injury Classification, *CIC* Chan et al. Injury Classification

Statistically significant differences in inter-reader reliability were observed between MCIC SUMMARY and CIC SUMMARY (*p* < 0.001), as well as between BAMIC SUMMARY and CIC SUMMARY (*p* < 0.001). There was no statistically significant difference in inter-reader reliability between BAMIC SUMMARY and MCIC SUMMARY (*p* = 0.109; see Table 4 for details).

**Table 4:** Differences between inter-reader values of classification systems (showing p-values, z-testing). **BOLD** numbers indicate statistical significance (*p* < 0.05)

	BAMIC_ SUMMARY	BAMIC_ Severity	BAMIC_ Location	MCIC_ SUMMARY	CIC_ Severity	CIC_ Anatomical Location	CIC_ Muscle Main Location	CIC_ Muscle Sublocation	CIC_ SUMMARY
BAMIC_SUMMARY		** < 0.001**	0.564	0.109	**0.009**	**0.032**	**0.007**	**< 0.001**	**< 0.001**
BAMIC_Severity	**< 0.001**		**< 0.001**	**0.002**	**0.008**	**0.002**	**< 0.001**	**< 0.001**	**< 0.001**
BAMIC_Location	0.564	**< 0.001**		0.348	0.093	0.201	**0.010**	**< 0.001**	**< 0.001**
MCIC_SUMMARY	0.109	**0.002**	0.348		0.547	0.819	**0.001**	**< 0.001**	**< 0.001**
CIC_Severity	**0.009**	**0.008**	0.093	0.547		0.683	**< 0.001**	**< 0.001**	** < 0.001**
CIC_Anatomical_Location	**0.032**	**0.002**	0.201	0.819	0.683		< **0.001**	**< 0.001**	**< 0.001**
CIC_Muscle_Main_Location_1	**0.007**	**< 0.001**	**0.010**	**0.001**	**< 0.001**	**< 0.001**		**0.009**	**< 0.001**
CIC_Muscle_Sublocation	**< 0.001**	**< 0.001**	**< 0.001**	**< 0.001**	**< 0.001**	**< 0.001**	**0.009**		**0.043**
CIC_SUMMARY	**< 0.001**	**< 0.001**	**< 0.001**	**< 0.001**	**< 0.001**	**< 0.001**	**< 0.001**	**0.043**	

*BAMIC* British Athletics Muscle Injury Classification, *MCIC* Munich Consensus Injury Classification, *CIC* Chan et al. Injury Classification

### Severity and location of injuries

**Fig. 5:**
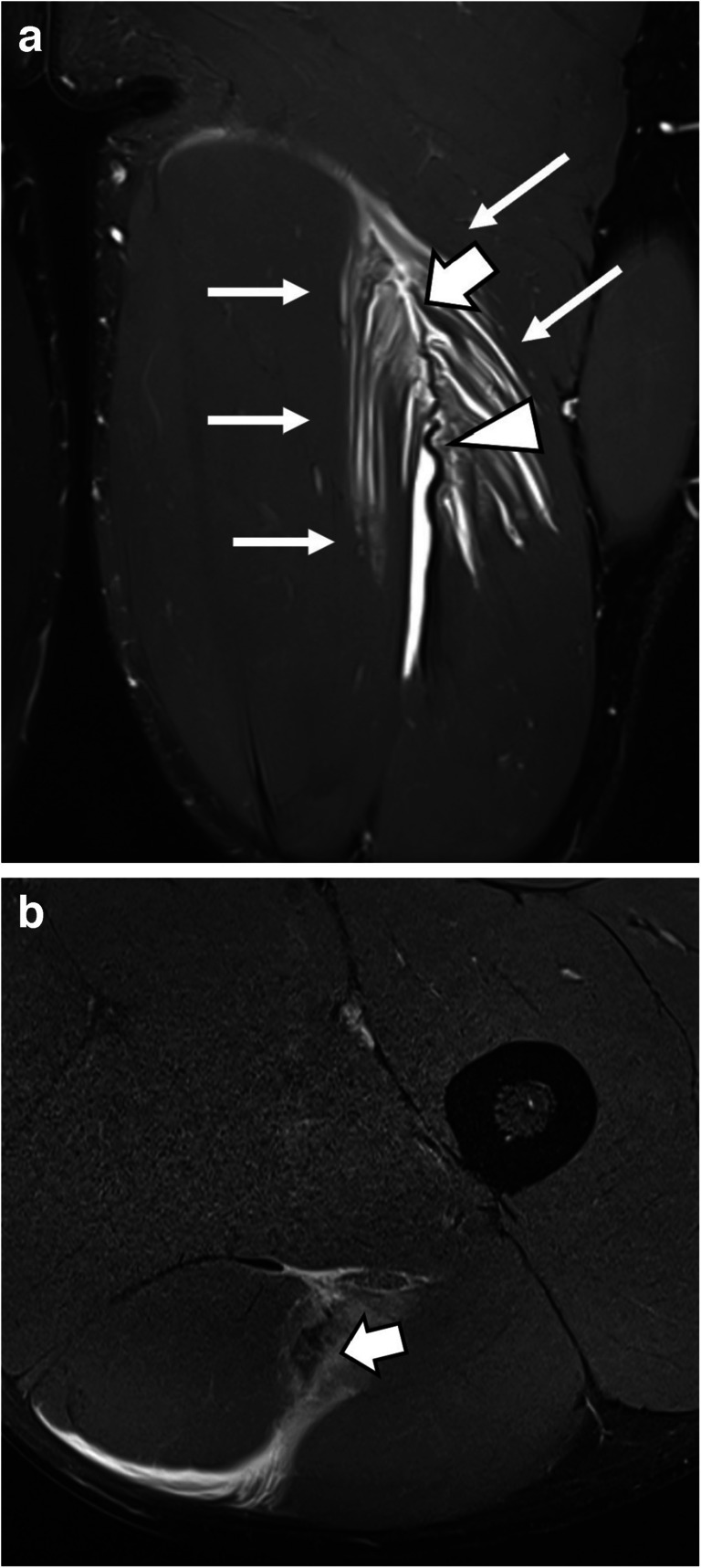
24-year-old male, soccer player, acute injury of left biceps femoris muscle (BAMIC: 4c, MCIC: 4, Chan: III.2.A.e (combined: myofascial and myotendinous; scored by reader 1). Coronal PDfs sequence (**A**) shows large edema (thin arrows; length: 14 cm) in the left biceps femoris muscle. Thick arrow and arrow head show the torn, elongated central tendon. Transverse PDfs sequence (**B**) shows the torn central tendon (arrow) and both myofascial and myotendinous edema

**Fig. 6:**
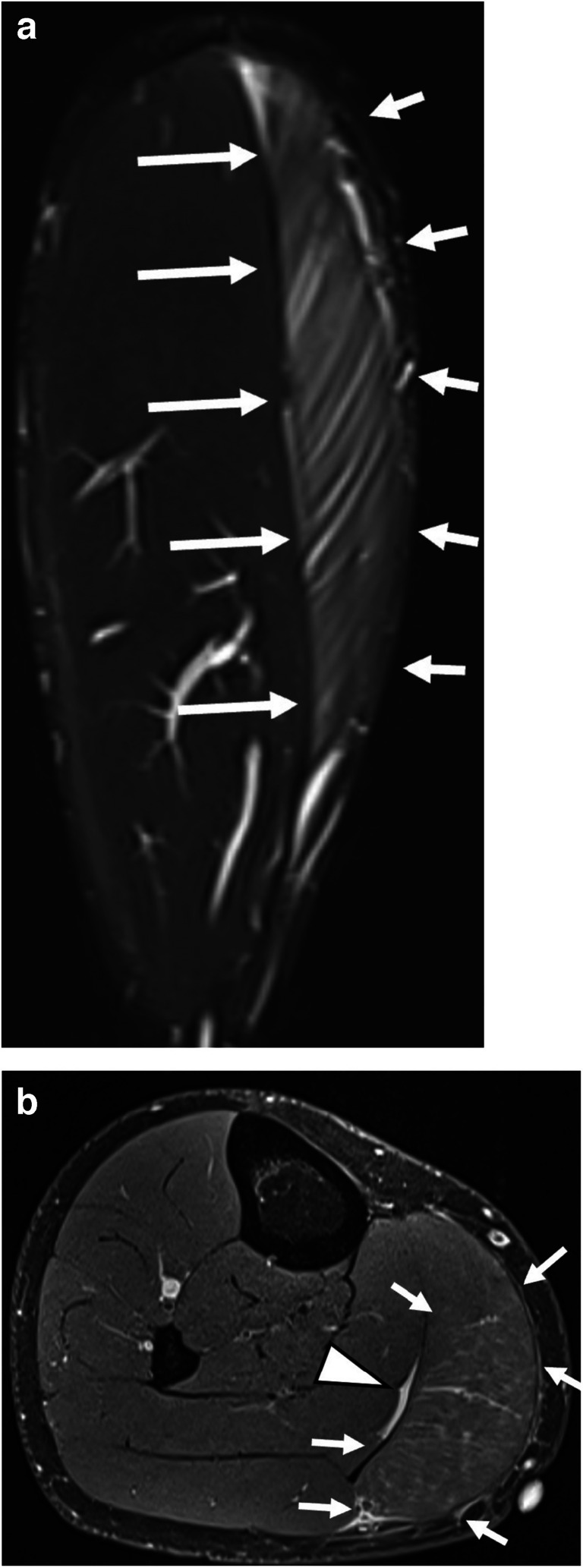
21-year-old male, acute injury of medial gastrocnemius head after a sprint (BAMIC: 3b; MCIC: 3B; CIC: II.2.B.e, scored by reader 1). Coronal STIR sequence (**A**) shows large edema (arrows, length: 24 cm) in the medial gastrocnemius muscle. Transverse PDfs (**B**) reveals edema intramuscular (arrows; in more than 50% of the cross-sectional area) and in myofascial/perifascial location. Fluid (arrow head) is seen between the medial gastrocnemius and soleus muscle. This injury is also known as “tennis leg”

The following scores assigned by reader 1 are presented to contextualize the distribution of injury severity within the study cohort (for details, see Table 5). Most injuries were classified as Grade 2b (*n* = 31, 27.9%) in the BAMIC classification. In the MCIC, indirect muscle injuries Grade 3B (*n* = 52, 46.8%) were the most frequently assigned category. Using the CIC, the most frequently assigned ratings were severity Grade II (*n* = 76, 68.5%) and location in the muscle belly (muscle main location type 2 = 57.7%). Among muscle lesions, the main locations were the middle (type B = 25.2%) and distal (type C = 20.7/) parts of the muscle. For the sublocation of muscle injuries, the myotendinous zone (type d = 19.8%) was the most common. Examples of muscle injury scoring are presented in Figs. 5 and 6.

**Table 5:** Detailed grading of reader 1: demonstrating injury patterns in the study cohort

	All													
BAMIC	111	Ia	Ib	Ic	IIa	IIb	IIc	IIIa	IIIb		IIIc	IV		IVc
		2 (1.8%)	8 (7.2%)	-	14 (12.6%)	31 (27.9%)	5 (4.5%)	8 (7.2%)	11 (9.9%)		8 (7.2%)	1 (0.9%)		23 (20.7%)
		I 9.1%			II 45%			III 24.3%				IV 21.6%		
MCIC	111	3A		3B		4								
		35 (31.6%)		52 (46.8%)		24 (21.6%)								
CIC Severity	111	I		II		III								
		12 (10.8%)		76 (68.5%)		23 (20.7%)								
CIC Anatomical location	111	1 Proximal MTJ		2 Muscle		3 Distal MTJ								
		30 (27.0%)		64 (57.7%)		17 (15.3%)								
CIC Muscle Main Location	64	A Proximal		B Middle		C Distal								
		13 (11.7%)		28 (25.2%)		23 (20.7%)								
CIC Muscle Sublocation	64	a intramuscular		B myofascial		c myofascial/perifascial		d myotendinous		e combined				
		6 (5.4%)		4 (3.6%)		15 (13.5%)		22 (19.8%)		17 (15.3%)				

*BAMIC*, British Athletics Muscle Injury Classification, *MCIC*, Munich Consensus Injury Classification, *CIC* Chan et al. Injury Classification

When looking at all cases across both BAMIC and CIC, the grading of injury severity demonstrated higher inter-reader agreement (*κ* = 0.594–0.696) compared to the assessment of injury location within the muscle (*κ* = 0.349–0.576).

### Thigh versus lower leg injuries

As shown in Table 2, all three classification SUMMARIES demonstrated an increase in inter-reader agreement when applied to thigh injuries compared to lower leg injuries. MCIC SUMMARY showed substantial inter-reader reliability for non-hamstring injuries and only moderate inter-reader reliability for hamstring injuries in the thigh.

CIC SUMMARY consistently exhibited the lowest inter-reader reliability, both for thigh and lower leg injuries. For assessment of thigh and lower leg injuries using the CIC SUMMARY, inter-reader reliability was highest for non-hamstring injuries (*κ* = 0.354; 95% CI = 0.345, 0.363) in the thigh.

Overall, the highest reproducibility was achieved for non-hamstring injuries assessed with the MCIC SUMMARY (*κ* = 0.749; 95% CI = 0.720–0.777), while the lowest was observed for lower leg injuries assessed with CIC SUMMARY (*κ* = 0.199; 95% CI = 0.185–0.213).

### Confidence of radiologists

Likert scale values for the BAMIC/MCIC/CIC were 4/5/3 for reader 1, 4/5/4 for reader 2, 4/4/3 for reader 3, and 4/5/3 for reader 4.

## Discussion

This study demonstrates a substantial difference in inter-reader reliability when comparing the three evaluated classification systems. Overall, both the British Athletics Muscle Injury Classification (BAMIC) and the Munich Consensus Injury Classification (MCIC) systems showed superior inter-reader agreement (moderate) compared to the Chan-Injury Classification (CIC), which exhibited only fair agreement. The overall low kappa value for CIC is primarily attributed to poor agreement regarding the main muscle injury location (*κ* = 0.429 [95% CI = 0.417–0.442]) and the muscle injury sublocation (*κ* = 0.349 [95% CI = 0.339–0.358]). Specifically, the CIC includes 3 main overall locations (proximal MTJ, muscle and distal MTJ), 3 main muscle locations (proximal, middle, distal), and 5 muscle sublocations, likely contributing to the reduced consistency among readers, whereas BAMIC includes only three locations.

Although some studies associate specific injury locations such as distal aponeurosis injuries of the rectus femoris muscle [22] or central aponeurosis injuries of the soleus muscle with longer return-to-play times, the evidence remains inconclusive. It is fundamentally unclear whether the anatomical intramuscular injury localization needs to be classified in as much detail as suggested by the CIC to ensure a clinically meaningful diagnosis.

Notably, inter-reader agreement in our study was substantially higher for injury severity grading (substantial for BAMIC and MCIC vs. moderate for CIC) than for injury localization (moderate for BAMIC vs. fair for CIC). These findings are in line with results reported by Wangensteen et al. [23], who assessed the inter-reader reliability of the CIC and BAMIC systems in 45 acute muscle injuries. They observed “almost perfect” agreement (*κ* = 0.81–1.0) when evaluating injury severity alone. However, inter-reader reliability varied substantially when grading intramuscular subcategories, ranging from fair to almost perfect. Interestingly, the overall kappa values for inter-reader agreement for both CIC and BAMIC in their study were slightly higher than those in our cohort. This difference is likely attributable to the inclusion of four readers in our study compared to only two in theirs, which inherently increases variability and potentially enhances statistical stability. Furthermore, Wangensteen et al. focused on hamstring injuries only, while our study cohort included other muscle groups as well. Part of both the CIC and BAMIC is anatomic localization, and the muscle anatomy varies depending on the muscle being assessed. The severity of muscle injuries in our study cohort was greater than that reported by Wangensteen et al.[23], based on a comparison of BAMIC classifications assigned by reader 1 in both studies. While most injuries were classified as grade II in both cohorts (45% in our study vs. 59% in Wangensteen et al.), nearly one-quarter of lesions in our cohort were graded as grade III (24% vs. 19.6%), and 21.6% were classified as grade IV (vs. none in the Wangensteen study). Due to the retrospective design of our study, with case selection based on the presence of muscle injuries described in the summary of radiology reports, no grade 0 lesions were identified (compared to 13.7% in the prospective cohort of Wangensteen et al.).

Interestingly, our study showed slightly higher inter-reader reliability for thigh injuries than for lower leg injuries, which may be attributed to the larger size of the thigh muscles and their more distinct anatomical landmarks. Within the thigh, we observed slightly better reliability for non-hamstring injuries compared to hamstring muscle injuries. Among the non-hamstring injuries in our study, the rectus femoris muscle was the most frequently affected, which is consistent to previous studies [6, 24].

There are no other studies comparing these three classification systems in a study cohort. Patel et al. [25] also reported excellent kappa values (*κ* = 0.80–0.88) when evaluating the inter-rater agreement of the BAMIC system, using two readers to assess 65 hamstring muscle injuries, thereby supporting its reliability and reproducibility.

To date, there are no other data available evaluating the inter-rater reliability of the MCIC and the CIC system. The MCIC system assesses injury severity independently of injury location and includes only three categories of muscle injuries, in contrast to BAMIC (11 subcategories) and CIC (up to 14 subcategories), which likely contributes to its higher inter-reader reliability. Furthermore, the principles and assumptions underlying the MCIC system are not universally accepted [26]. For non-contact muscle injuries, only minor and moderate partial tears (types 3 A and 3B) are taxonomically differentiated from subtotal muscle tears or tendinous avulsions (type 4) [19]. However, the MCIC system does not distinguish the specific tissue involved or the precise location within the muscle. Inter-reader disagreements may arise due to the lack of detailed guidance within the classification.

Radiologists’ confidence when using the classifications was highest for the MCIC and lowest for the Chan classification (CIC).

### Limitations

This study was performed retrospectively, and we had no access to clinical patient data. All readers were experienced and fellowship-trained in musculoskeletal (MSK) radiology. Therefore, the results may not be generalizable to non-specialized radiologists.

The classification systems exhibit limited comparability with respect to their structural complexity with BAMIC and CIC being more complex than the MCIC. We focused only on three classification systems routinely used by our referrers, excluding systems limited to specific muscle groups (such as the MLG-R classification system for hamstring injuries [27]). The assessment for inter-reader reliability for injury location could only be done for BAMIC and CIC, since the MCIC does not include injury location. Further research is needed to evaluate the excluded classification systems.

## Conclusion

Overall, the MCIC and BAMIC systems demonstrate moderate, and the CIC fair inter-reader reliability. The challenge of classifications appears to lie mainly in the precise anatomical injury localization, less in the grading of injury severity. The highest reproducibility was observed for non-hamstring injuries in the thigh using the MCIC, while the lowest was seen for lower leg injuries using the CIC.

As the division into multiple anatomical subcategories was recorded, a substantial difference in the performance of each classification system could be observed when subcategories were compared. It can be hypothesized that selecting the most appropriate classification system for a specific anatomical subcategory of a given lesion would considerably enhance inter-reader agreement. Further investigation is necessary to validate this hypothesis.

All procedures performed in studies involving human participants were in accordance with the ethical standards of the institutional and/or national research committee and with the 1964 Helsinki declaration and its later amendments or comparable ethical standards.

Supplementary information.

## Supplementary Information

Below is the link to the electronic supplementary material.

ESM 1 Supplementary Material 1 (DOCX 15.3 KB)

## Data Availability

The data that support the findings of this study are not openly available due to reasons of sensitivity and are available from the corresponding author upon reasonable request.

## References

[1] Edouard P, Branco P, Alonso JM. Muscle injury is the principal injury type and hamstring muscle injury is the first injury diagnosis during top-level international athletics championships between 2007 and 2015. Br J Sports Med. 2016;50(10):619–30. 26887415 10.1136/bjsports-2015-095559

[2] Ekstrand J, Hagglund M, Walden M. Epidemiology of muscle injuries in professional football (soccer). Am J Sports Med. 2011;39(6):1226–32. 21335353 10.1177/0363546510395879

[3] Orchard JW, Seward H, Orchard JJ. Results of 2 decades of injury surveillance and public release of data in the Australian Football League. Am J Sports Med. 2013;41(4):734–41. 23460329 10.1177/0363546513476270

[4] Elliott MC, Zarins B, Powell JW, Kenyon CD. Hamstring muscle strains in professional football players: a 10-year review. Am J Sports Med. 2011;39(4):843–50. 21335347 10.1177/0363546510394647

[5] Brooks JH, Fuller CW, Kemp SP, Reddin DB. Incidence, risk, and prevention of hamstring muscle injuries in professional rugby union. Am J Sports Med. 2006;34(8):1297–306. 16493170 10.1177/0363546505286022

[6] Ouellette H, Thomas BJ, Nelson E, Torriani M. MR imaging of rectus femoris origin injuries. Skeletal Radiol. 2006;35(9):665–72. 16738911 10.1007/s00256-006-0162-9

[7] Geiss Santos RC, Van Hellemnondt F, Yamashiro E, Holtzhausen L, Serner A, Farooq A, et al. Association between injury mechanisms and magnetic resonance imaging findings in rectus femoris injuries in 105 professional football players. Clin J Sport Med. 2022;32(4):e430–5. 34050059 10.1097/JSM.0000000000000935

[8] Kassarjian A, Rodrigo RM, Santisteban JM. Intramuscular degloving injuries to the rectus femoris: findings at MRI. AJR Am J Roentgenol. 2014;202(5):W475-480. 24450607 10.2214/AJR.13.10931

[9] Crema MD, Yamada AF, Guermazi A, Roemer FW, Skaf AY. Imaging techniques for muscle injury in sports medicine and clinical relevance. Curr Rev Musculoskelet Med. 2015;8(2):154–61. 25708212 10.1007/s12178-015-9260-4 PMC4596169

[10] Chang JS, Kayani B, Plastow R, Singh S, Magan A, Haddad FS. Management of hamstring injuries: current concepts review. Bone Joint J. 2020;102-B(10):1281–1288. 10.1302/0301-620X.102B10.BJJ-2020-1210.R1 32993323

[11] Heiss R, Tol JL, Pogarell T, Roemer FW, Reurink G, Renoux J, et al. Imaging of muscle injuries in soccer. Skeletal Radiol. 2025;54(4):655–67. 37991553 10.1007/s00256-023-04514-1

[12] Hirahata Y, Yasui Y, Sasahara J, Inui T, Nakagawa T, Kawano H, Miyamoto W. Role of ultrasonography and MRI in acute hamstring strains: diagnostic and prognostic insights. Diagnostics (Basel). 2025. 10.3390/diagnostics15091053. 10.3390/diagnostics15091053 PMC12071714 40361871

[13] Moen MH, Reurink G, Weir A, Tol JL, Maas M, Goudswaard GJ. Predicting return to play after hamstring injuries. Br J Sports Med. 2014;48(18):1358–63. 25037199 10.1136/bjsports-2014-093860

[14] Jacobsen P, Witvrouw E, Muxart P, Tol JL, Whiteley R. A combination of initial and follow-up physiotherapist examination predicts physician-determined time to return to play after hamstring injury, with no added value of MRI. Br J Sports Med. 2016;50(7):431–9. 26843538 10.1136/bjsports-2015-095073

[15] Wangensteen A, Almusa E, Boukarroum S, Farooq A, Hamilton B, Whiteley R, et al. MRI does not add value over and above patient history and clinical examination in predicting time to return to sport after acute hamstring injuries: a prospective cohort of 180 male athletes. Br J Sports Med. 2015;49(24):1579–87. 26305004 10.1136/bjsports-2015-094892

[16] Guermazi A, Roemer FW, Robinson P, Tol JL, Regatte RR, Crema MD. Imaging of muscle injuries in sports medicine: sports imaging series. Radiology. 2017;285(3):1063. 29155622 10.1148/radiol.2017174038

[17] Ekstrand J, Askling C, Magnusson H, Mithoefer K. Return to play after thigh muscle injury in elite football players: implementation and validation of the Munich muscle injury classification. Br J Sports Med. 2013;47(12):769–74. 23645834 10.1136/bjsports-2012-092092 PMC3717808

[18] Pollock N, James SL, Lee JC, Chakraverty R. British athletics muscle injury classification: a new grading system. Br J Sports Med. 2014;48(18):1347–51. 25031367 10.1136/bjsports-2013-093302

[19] Mueller-Wohlfahrt HW, Haensel L, Mithoefer K, Ekstrand J, English B, McNally S, et al. Terminology and classification of muscle injuries in sport: the Munich consensus statement. Br J Sports Med. 2013;47(6):342–50. 23080315 10.1136/bjsports-2012-091448 PMC3607100

[20] Chan O, Del Buono A, Best TM, Maffulli N. Acute muscle strain injuries: a proposed new classification system. Knee Surg Sports Traumatol Arthrosc. 2012;20(11):2356–62. 22773066 10.1007/s00167-012-2118-z

[21] Landis JR, Koch GG. The measurement of observer agreement for categorical data. Biometrics. 1977;33(1):159–74. 843571

[22] Shimozaki K, Nakase J, Asai K, Yoshimizu R, Kimura M, Kanayama T, et al. Relationship between anatomical injury site of rectus femoris muscle strain and time taken to return to play in Japanese professional soccer players. J Orthop Surg (Hong Kong). 2022;30(3):10225536221141786. 36548509 10.1177/10225536221141786

[23] Wangensteen A, Tol JL, Roemer FW, Bahr R, Dijkstra HP, Crema MD, et al. Intra- and interrater reliability of three different MRI grading and classification systems after acute hamstring injuries. Eur J Radiol. 2017;89:182–90. 28267537 10.1016/j.ejrad.2017.02.010

[24] Katagiri H, Forster BB, Engebretsen L, An JS, Adachi T, Saida Y, et al. Epidemiology of MRI-detected muscle injury in athletes participating in the Tokyo 2020 Olympic Games. Br J Sports Med. 2022;57(4):218–24. 36588405 10.1136/bjsports-2022-105827 PMC9933160

[25] Patel A, Chakraverty J, Pollock N, Chakraverty R, Suokas AK, James SL. British athletics muscle injury classification: a reliability study for a new grading system. Clin Radiol. 2015;70(12):1414–20. 26385202 10.1016/j.crad.2015.08.009

[26] Fontanier V, Bruchard A, Tremblay M, Mohammed R, da Silva-Oolup S, Suri-Chilana M, et al. Classification of myo-connective tissue injuries for severity grading and return to play prediction: a scoping review. J Sci Med Sport. 2025;28(1):46–55. 39232948 10.1016/j.jsams.2024.07.016

[27] Valle X, Mecho S, Alentorn-Geli E, Jarvinen TAH, Lempainen L, Pruna R, et al. Return to play prediction accuracy of the MLG-R classification system for hamstring injuries in football players: a machine learning approach. Sports Med. 2022;52(9):2271–82. 35610405 10.1007/s40279-022-01672-5

