# Supplementary material for: Classification systems for assessing acute muscle injuries: a retrospective comparison of inter-reader agreements
Source: Skeletal Radiol. 2025 Aug 13;55(1):191–203. doi: 10.1007/s00256-025-04988-1 (PMC12627197; doi:10.1007/s00256-025-04988-1)
Supplement: Supplementary file 1 — Supplementary Material 1 (DOCX 15.3 KB) [file 256_2025_4988_MOESM1_ESM.docx]

Table S1: Routine protocol for native 3T-MRI (VIDA, Siemens) for muscle injuries in our institution

| Sequence | FoV  (mm) | Slice thickness  (mm) | TE  (ms) | TR  (ms) |
| --- | --- | --- | --- | --- |
| Coronal STIR | 380 | 4 | 38 | 6490 |
| Transverse T1 | 179 | 4 | 22 | 568 |
| Sagittal PDfs | 350 | 3 | 34 | 4570 |
| Coronal PDfs | 350 | 3 | 47 | 4260 |
| Transverse PDfs | 179 | 4 | 44 | 460 |

Note: FoV= field of view; TE=echo time; TR=repetition time; ms= milliseconds; STIR=Short-Tau Inversion Recovery; PDfs= proton density fat saturated.
